# Task-specific versus free-choice non-driving-related tasks: Enhancing automated driving takeover research through varied task engagement

**DOI:** 10.1016/j.isci.2025.112783

**Published:** 2025-05-29

**Authors:** Wenjun Wang, Zhenyuan Wang, Qingkun Li, Yizi Su, Chao Zeng, Bo Cheng

**Affiliations:** 1 State Key Laboratory of Automotive Safety and Energy, School of Vehicle and Mobility, Tsinghua University, Beijing 100084, China; 2 Beijing Key Laboratory of Human–Computer Interaction, Institute of Software, Chinese Academy of Sciences, Beijing 100190, China; 3 Automotive Software Innovation Center (Chongqing), Chongqing 401331, China; 4 College of Information Science and Engineering, Henan University of Technology, Zhengzhou 450001, China; 5 Hami Vocational and Technical College, Hami 839001, China

**Keywords:** Applied sciences, Computer science, Artificial intelligence

## Abstract

Level 3 automated driving systems require drivers to occasionally retake control, making it crucial to understand factors influencing takeover performance. This study investigates how single versus multiple non-driving-related tasks (NDRTs) affect driver behavior, perceptions, and takeover capability. Using a high-fidelity driving simulator with 32 participants, conditions varying NDRT types and task-switching frequency were examined. Results indicate that diverse NDRT engagement and frequent task switching significantly influence takeover performance. Moreover, our decision tree models highlighted the number of NDRT types and task-switching frequency as critical predictors of driver response effectiveness. These findings underscore the importance of accounting for task engagement diversity and switching behaviors in automated vehicle system design. This study provides practical implications for optimizing human-automation interactions and enhancing the safety of automated driving systems by managing drivers’ attentiveness for their in-cabin activities.

## Highlights

• Type of non-driving-related tasks affects drivers’ behaviors in automated driving
• Trial setting moderated the impact of task engagement on takeover performance
• Task switching should be considered as a factor for takeover research

## Graphical abstract

**Figure:**
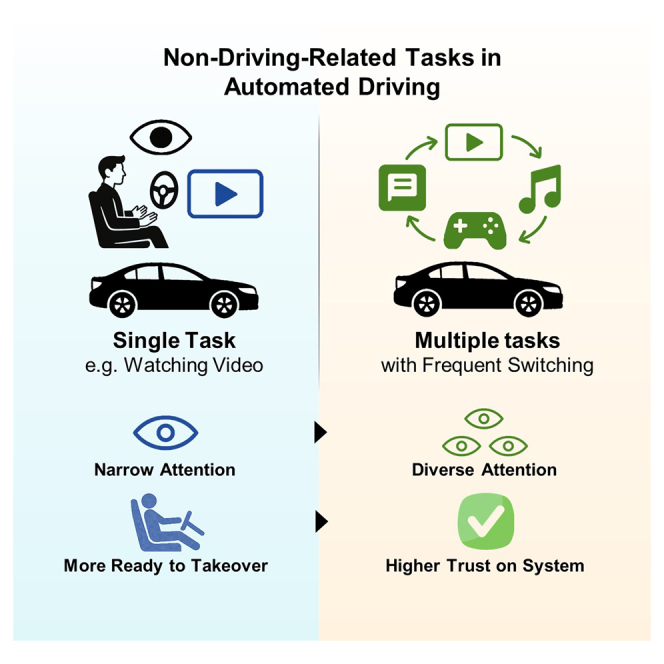

## Introduction

Automated driving technology is rapidly transforming the transportation landscape and is poised to revolutionize the way we travel.^1^ As vehicular technologies and digitalization trends continue to evolve, there is a growing recognition of the potential impacts of automated driving on various aspects of behavior inside vehicles, including the ability to engage in non-driving-related tasks (NDRTs).^2^^,^^3^ Although NDRTs can enhance the driving experience by allowing drivers to multitask, this technological advancement also brings challenges, particularly in the safe and efficient transfer of control between the automated driving system and the human driver, a process referred to as takeover.^4^^,^^5^

Takeover presents potential risks, especially when the human driver is engaged in an NDRT before and during it.^6^ Ensuring driver situation awareness, readiness, and engagement is thus crucial for the successful transition between automated and manual driving modes, as emphasized by the World Forum for the Harmonization of Vehicle Regulations.^7^ As level 3 (L3) automated vehicles become more available, addressing these challenges will be essential for maximizing safety and efficiency in the evolving transportation landscape.^8^

### Task-specific versus free-choice non-driving-related tasks in takeover performance

In recent years, numerous studies have investigated the effects of NDRTs on takeover performance, with the majority utilizing driving simulators to emulate driving conditions.^9^^,^^10^^,^^11^ However, the extent to which these experimental setups mirror real-world driving scenarios remains a critical consideration. A key aspect is whether the NDRTs employed in studies reflect the diversity and spontaneity of tasks drivers perform in actual automated driving environments.

Traditionally, research has focused on task-specific NDRTs, such as the N-back test or visual tracking tasks, chosen for their ease of manipulation and control over task demands.^12^^,^^13^^,^^14^ Although these standardized tasks facilitate controlled experimentation, they may not fully capture the complexity and authenticity of real-world driver behaviors in automated driving scenarios.^15^ Consequently, the generalizability of findings from such studies to everyday driving situations is limited.

To address these limitations, some studies have incorporated naturalistic NDRTs—tasks that drivers are more likely to perform in real automated driving contexts, such as browsing social media, making phone calls, or using navigation apps.^4^^,^^16^^,^^17^ These tasks are inherently more complex, involving multiple cognitive processes like attention, working memory, and decision-making.^18^ For instance, reading requires visual processing and comprehension skills, whereas texting involves manual dexterity and visual-manual distraction.^19^ Additionally, naturalistic NDRTs vary in cognitive demand and duration, reflecting the unpredictability and multitasking nature of real-world automated driving scenarios.

Despite the incorporation of naturalistic NDRTs, existing studies often impose predefined task content, limiting participants’ freedom to choose or switch tasks as they would in real-life situations.^20^^,^^21^ This restriction can lead to differences in attention levels and task engagement, creating discrepancies between experimental conditions and actual driving environments. Moreover, many studies confine participants to a single NDRT per trial, which diverges significantly from real-world conditions where drivers frequently switch between multiple tasks.^22^^,^^23^ Such constraints undermine the ability to accurately simulate the multitasking demands faced by drivers in automated vehicles.

Therefore, there is a pressing need to explore how task-specific and free-choice NDRTs—where drivers can select and switch between multiple tasks—affect takeover performance. Understanding these dynamics is crucial for developing more realistic and applicable insights into driver behavior, ultimately contributing to the design of safer and more efficient automated driving systems.

### Research aim

This study aims to deepen the understanding of how different types of NDRTs—specifically task-specific and free-choice engagements—affect takeover performance in L3 automated driving. It contributes to the field in several key areas. First, it compares task-specific and free-choice trials, offering insights into how varying task engagement affects takeover performance and driver behavior in driving simulator studies. Second, it examines the impact of task-switching behaviors on takeover performance, an area not extensively explored in previous works. This approach provides a more comprehensive perspective of drivers’ behaviors and subjective evaluations during takeovers in L3 automated driving, introducing nuanced factors and considerations not previously considered in the design and execution of simulator studies. The findings underscore the importance of examining trial settings and other factors on takeover scenarios, with far-reaching implications for future research, experimental design, and the evolution of automated driving systems.

### Structure of the paper

**Figure 1:**
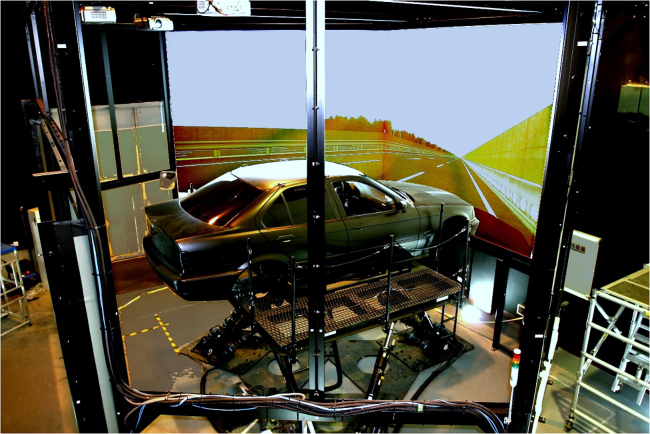
Driving simulator at Tsinghua University

**Figure 2:**
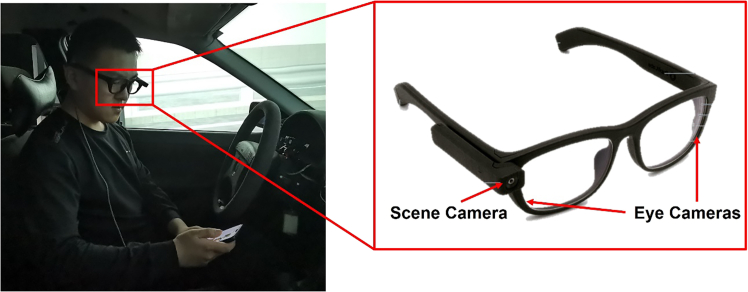
Pupil invisible eye tracker with NDRTs

**Figure 3:**
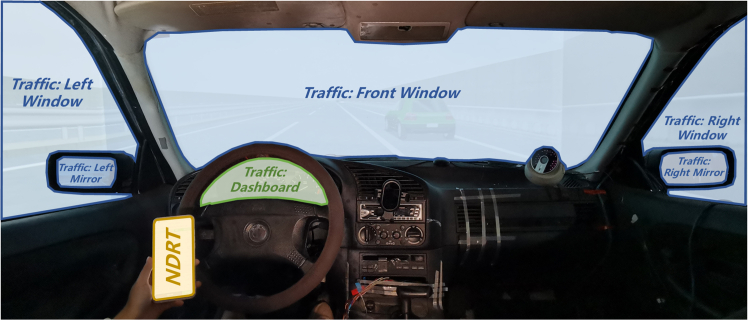
Visualization of AOIs in the driving simulator environment

**Figure 4:**
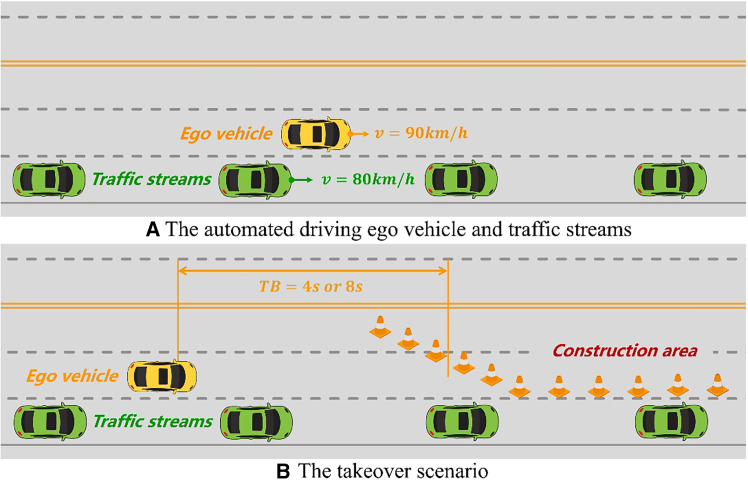
Takeover scenarios’ parameters and time budgets

**Figure 5:**
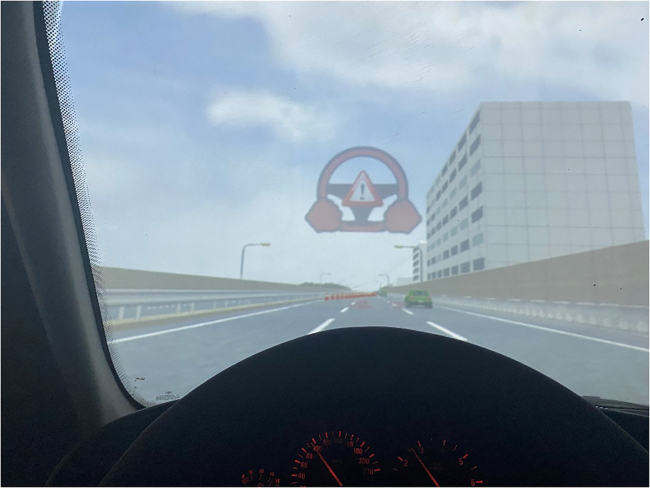
Visual representation of takeover request issuance

**Figure 6:**
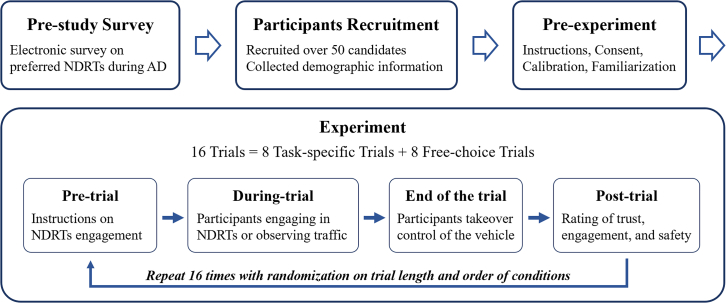
Flowchart of study procedure

The remainder of this paper is organized as follows: Results section presents the experimental results and main findings. Discussion section discusses the results presented in results and concludes this work by synthesizing the key insights and contributions of the study in terms of improving the realism and applicability of driving simulator research. Methods section introduces the apparatus (see Figure 1, 2 and 3), experimental scenarios (see Figure 4), experimental design (see Figure 5), participants, and procedure (see Figure 6).

## Results

### Driver attention

The impact of NDRT setting (i.e., free-choice vs. task-specific) on various aspects of driver behavior was examined in this analysis. These aspects included engagement in different types of NDRTs measured by NNDRTT, fixations on traffic measured by NT, and proportion of engagement in NDRTs measured by PNDRT, as descripted in Table 1.

**Table 1:** Eye-tracking-based indicators and self-evaluation measures

Variable	Description
NNDRTT	the total number (count) of distinct NDRT types that participants engaged with in the 20-s window before the TOR
NT	the total number (count) of fixations on traffic-related stimuli (e.g., vehicles, traffic signals) within the 20-s window
PNDRT	the ratio (dimensionless, 0–1) of fixations on NDRTs relative to the total number of fixations in the 20-s window
NSBNDRTs	the total number (count) of times participants switched between different NDRTs in the 20-s period
TLS	the timestamp (seconds) of the last switch between NDRTs before the TOR
ADST	a subjective rating (scale 1–100) reflecting participants’ trust in the automated driving system
SNE	a subjective rating (scale 1–100) of how engaged participants felt with the NDRT during the drive
SRT	a subjective rating (scale 1–100) of how ready participants felt to take over vehicle control at the TOR

Given that our preliminary normality checks indicated that these data did not meet the assumptions of a parametric test, we selected the Mann-Whitney U test to compare these variables between the two trial types. The Mann-Whitney U test is a nonparametric alternative that is robust to violations of normality and is suitable for ordinal or skewed data distributions.

These results revealed significant differences in all three aspects of driver behavior, as shown in Table 2. Specifically, drivers in the free-choice trials tended to engage in a greater variety of NDRTs (median NNDRTT in free-choice trials = 2, median NNDRTT in task-specific trials = 1, *U* = 41184.5, *p* < 0.001), were less likely to fixate on traffic (median NT in free-choice trials = 0, median NT in task-specific trials = 1, *U* = 27547.0, *p* = 0.004), and were more fully engaged in NDRTs (median PNDRT in free-choice trials = 1.000, median PNDRT in task-specific trials = 0.947, *U* = 38832.0, *p* < 0.001).

**Table 2:** Summary of driver behavior differences between free-choice and task-specific trials

Aspect of driver’s behavior	Median value in free-choice trials	Median value in task-specific trials	Mann-Whitney U test	*p* value
NNDRTT	2	1	41184.5	<0.001
NT	0	1	27547.0	0.004
PNDRT	1.000	0.947	38832.0	<0.001

**Figure 7:**
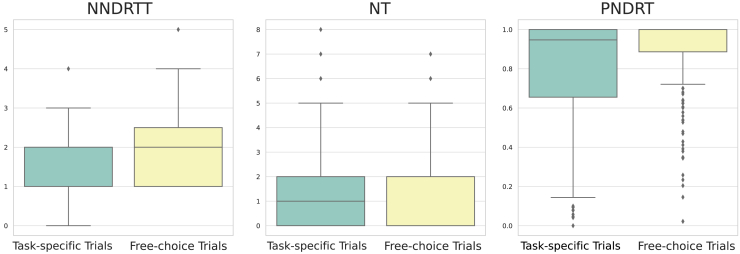
Distribution of driver behavior aspects in free-choice and task-specific trials

These findings are illustrated in Figure 7, which shows the distributions of NNDRTT, NT, and PNDRT across both trial types. Free-choice trials were characterized by a greater spread of values in NNDRTT and PNDRT and fewer NTs, reflecting the greater complexity and variability of these trials compared to task-specific trials.

These results suggest that the type of NDRT setting significantly affects drivers’ behaviors in automated driving takeover experiments. Specifically, drivers in free-choice trials exhibit more diverse and flexible behavior, mirroring the greater diversity of real-world driving scenarios.

### Subjective ratings

The impact of trial type on selected subjective ratings was assessed, including automated driving system trust (ADST), subjective NDRT engagement (SNE), and subjective readiness for takeover (SRT), each scored on a scale from 0 to 100, as descripted in Table 1. Similar to the objective measures of driver behavior, our normality checks indicated that these subjective ratings were also not normally distributed. Therefore, we used the same Mann-Whitney U test to compare these variables between the two trial types, ensuring consistency and robustness across all measures despite the lack of normality.

The results of this analysis revealed significant differences in all three evaluated ratings, as shown in Table 3. Drivers in the free-choice trials demonstrated greater trust in the automated driving system (median ADST in free-choice trials = 78, median ADST in task-specific trials = 71, *U* = 37384.5, *p* = 0.001) and greater engagement in NDRTs (median SNE in free-choice trials = 84, median SNE in task-specific trials = 80, *U* = 39222.5, *p* < 0.001). However, they showed lower readiness to take over (median SRT in free-choice trials = 52, median SRT in task-specific trials = 62, *U* = 25669.5, *p* < 0.001).

**Table 3:** Summary of subjective evaluated rating differences in free-choice and task-specific trials

Rating aspect	Median value in free-choice trials	Median value in task-specific trials	Mann-Whitney U	*p* Value
ADST	78	71	37384.5	0.001
SNE	84	80	39222.5	<0.001
SRT	52	62	25669.5	<0.001

**Figure 8:**
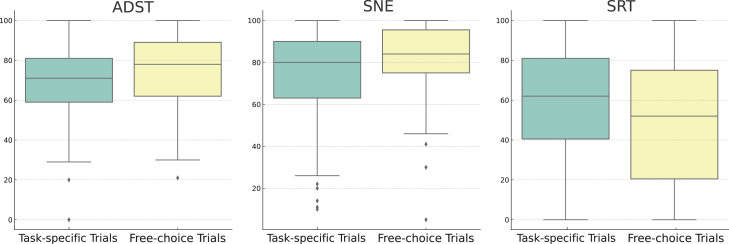
Distribution of subjective rating aspects in free-choice and task-specific trials

These findings are illustrated in Figure 8, which presents the distributions of subjective evaluated ratings in both trial types. The free-choice trials were characterized by higher ADST and SNE and lower SRT, reflecting the more dynamic and complex nature of these trials compared to task-specific trials. These results provide clear evidence that trial type significantly impacts subjective ratings in automated driving takeover experiments, showing that drivers’ experiences and perceptions can vary significantly depending on the NDRT setting of the trial.

### The moderation effects of trial types

The relationship between the proportion of NDRT engagement and objective takeover performance metrics has been explored in previous studies.^24^^,^^25^^,^^26^^,^^27^ However, whether this relationship is influenced by the nature of drivers’ NDRTs (i.e., switching among multiple tasks freely or performing a single task specifically) remains unexplored. To shed light on this issue, moderation analyses were conducted on the potential moderating role of trial type in the relationship between NDRT engagement and objective takeover performance.

A nonparametric bootstrapped regression-based moderation analysis with 5,000 bootstrap samples was employed to examine the moderating effects. This method is particularly suitable for handling nonnormally distributed data. In this procedure, samples were drawn with replacement from the original dataset to generate an empirical distribution of the moderating effect estimates. For each bootstrap iteration, the relationship between the independent variable (PNDRT) and the dependent variables (various objective takeover performance metrics) was recalculated, incorporating trial type (task-specific vs. free-choice) as the moderating variable. The resulting distribution of estimates allowed us to derive robust 95% confidence intervals using the percentile method. This approach ensured that our estimates were reliable despite the nonnormality of the data.

The analyses revealed a significant moderating effect of trial type on the relationship between NDRT engagement and various objective takeover performance metrics. Specifically, 15 pairs of variables were found to exhibit significant moderating effects, several of which are presented in Table 4.

**Table 4:** Moderating effects of trial type on NDRT engagement and takeover performance metrics

Independent variables	Dependent variables	Lower bound of the confidence interval	Upper bound of the confidence interval	Moderating effect	*p* value
PNDRT	mean lateral acceleration	0.001	0.003	significant	0.003
	mean yaw rate	0.015	0.091	significant	0.004
	collision	0.027	0.447	significant	0.017
	mean throttle	−0.002	0.152	significant	0.018
	mean longitudinal acceleration	0.001	0.030	significant	0.019
	eyes on road time	−0.111	0.462	not significant	0.121
	minimum time to collision	−0.575	2.007	not significant	0.110
	reaction time	−0.942	0.491	not significant	0.228

**Figure 9:**
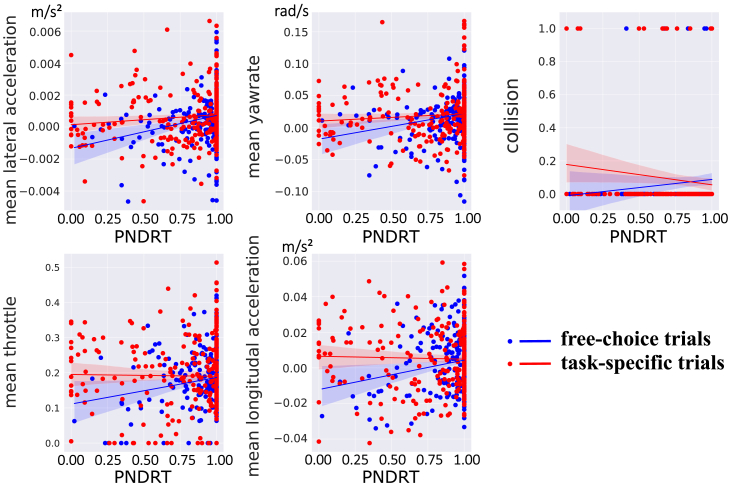
Visualization of the moderating effects of trial type on NDRT engagement and takeover performance metrics

The differential patterns in the relationship between NDRT engagement and objective takeover performance metrics during task-specific and free-choice trials are illustrated in Figure 9, where blue dots and lines represent the free-choice trials, and red dots and lines represent the task-specific trials. For instance, in task-specific trials, the relationships between PNDRT and mean lateral acceleration and between PNDRT and mean yaw rate were found to be weaker than those in the free-choice trials. This finding implies that trial type may influence how NDRT engagement impacts takeover performance.

Importantly, not all relationships were significantly moderated by trial type. Metrics such as eyes on road time (defined as the cumulative duration, measured in seconds, during which the driver’s gaze remains directed toward the roadway), reaction time, and minimum time to collision did not exhibit a significant moderating effect, suggesting that the relationships between NDRT engagement and these metrics are similar regardless of trial type.

### Impacts of NDRT engagement and task-switching behaviors on takeover performance

Addressing a gap in the literature on automated driving takeover performance, this analysis examined the impact of task-switching behaviors on takeover performance. While previous studies have focused on single-task scenarios, they have not encompassed the dynamics inherent in task-switching behavior—a crucial element of real-world driving. By incorporating multiple tasks, we investigated variables such as NNDRTT, NSBNDRTs, and TLS.

Decision tree regression models were employed to predict various takeover performance metrics using independent variables from both the experimental conditions and driver behavior groups, including TB, Repetition, NNDRTT, NSBNDRTs, and TLS. Decision tree regression was selected due to its suitability for datasets with non-normal distributions and mixed variable types—both characteristics present in our experimental data. Additionally, decision trees effectively model non-linear relationships without requiring strong assumptions about data distributions. Another advantage of decision trees is their intuitiveness and ease of interpretation, aligning closely with our study’s objective to clearly identify and understand key behavioral predictors influencing takeover performance. Furthermore, decision tree methods are particularly beneficial for analyses involving relatively small datasets.

**Figure 10:**
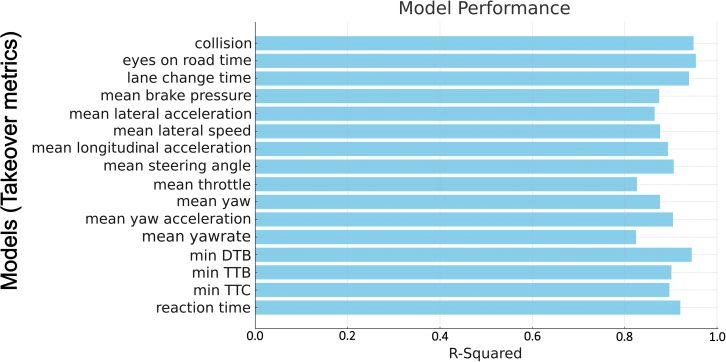
The performances of models in predicting takeover metrics

The models demonstrated robust performance, with several variables emerging as significant predictors across multiple models, as depicted in Figure 10. Specifically, the variables reflecting multi-tasking behaviors were identified as significant predictors in multiple models, suggesting that these variables, which capture the dynamics of task-switching behavior, significantly influence takeover performance.

For instance, TLS was found to be an important predictor in several models. This variable captures the time dynamics of drivers’ task-switching behavior, and our results suggest that it significantly influences takeover performance. Similarly, NNDRTT and NSBNDRTs, which reflect the variety and frequency of drivers’ engagement in NDRTs, were also found to be important predictors in several models. These findings thus underscore the impact of task-switching dynamics on takeover performance, which has been largely neglected in extant task-specific experimental designs.

**Figure 11:**
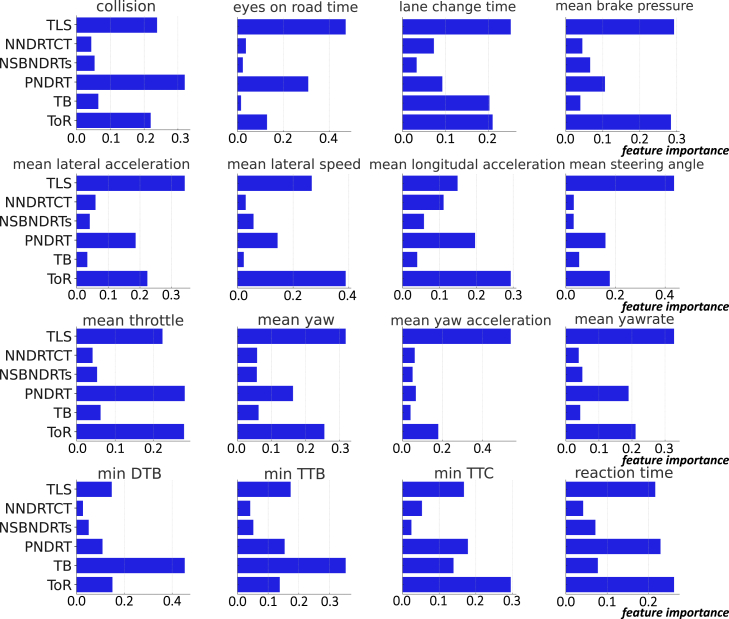
Feature importance for decision tree regression msodels

The impact of these variables is visualized in Figure 11, which illustrates the feature importance for each decision tree model. The aforementioned variables consistently emerged as important predictors across multiple models.

In summary, the results highlight the importance of considering task-switching behaviors in automated driving takeover experiments. By examining the dynamics of task-switching behavior, we identified significant predictors of takeover performance that were not observable in previous task-specific experimental designs. These findings underscore the complexity of drivers’ behavior during automated driving and the need for experimental designs that reflect this complexity.

## Discussion

This study investigated how two different trial settings—task-specific versus free-choice trials—impact driver behavior, subjective evaluations, and takeover performance in a driving simulator. The results indicate that free-choice trials, which allow participants to select and switch between multiple NDRTs, lead to more varied driver behaviors, distinct subjective evaluations, and a more complex interplay between NDRT engagement and takeover performance. Furthermore, the use of metrics capturing task-switching behaviors provides insights into predicting takeover performance.

### Variations in driver behavior and subjective evaluations across different trial settings

Our findings show that the trial setting significantly affects drivers’ behaviors. Research by Jaussein et al. points out that with highly automated driving, the driver’s cognitive focus can shift from distraction to active task engagement.^6^ Similarly, the free-choice setting in our study elicited a more diverse range of driver behaviors, aligning more closely with the complexities drivers might encounter when they have the freedom to choose their own activities. Previous work suggests that more flexible engagement with NDRTs can approximate the variety of conditions drivers may face in reality.^23^ Thus, incorporating free-choice trials enables a more comprehensive capture of driver behavior than limiting participants to predetermined tasks.

In addition to these behavioral differences, the trial setting influenced subjective evaluations. Drivers in free-choice trials reported higher trust in the automated driving system and greater NDRT engagement but lower readiness to take over. This pattern likely arises because free-choice scenarios encourage a relaxed attitude toward the vehicle’s automation and a higher cognitive load from managing multiple tasks. Studies have shown that when drivers perceive the system as providing more autonomous capabilities, they may attribute greater intelligence and trust to it.^28^^,^^29^ However, simultaneously, juggling multiple tasks can reduce the driver’s state of readiness to promptly resume control.

By comparing task-specific and free-choice conditions, we gain a more nuanced understanding of how different trial setups can shape driver trust, engagement, and takeover readiness. Such insights are valuable for designing experiments and automated driving systems that better account for the range of behaviors and perceptions drivers exhibit.

### Trial setting as a moderator of the relationship between NDRT engagement and takeover performance

Our findings also reveal that the relationship between NDRT engagement and takeover performance differs depending on the trial setting. Certain NDRTs displayed a stronger association with takeover outcomes in free-choice trials than in task-specific ones. This difference may arise because free-choice scenarios present drivers with a more complex cognitive landscape as they manage multiple tasks simultaneously—challenges that can affect the speed, accuracy, and efficiency of their takeover responses.^30^

Such results suggest that previous conclusions drawn solely from task-specific settings may not fully translate to more varied conditions. Free-choice trials can uncover intricate dynamics that are masked when drivers are restricted to a single, uniform task. This highlights the importance of considering how the trial setup might shape the interplay between NDRT engagement and takeover performance, ensuring that research findings remain relevant beyond tightly controlled experimental parameters.^31^^,^^32^

### Predicting takeover performance through task-switching metrics

A key contribution of this study lies in the use of variables related to task-switching behaviors—such as TLS, NNDRTT, and NSBNDRTs—to predict takeover performance. While previous research often focused on single-task scenarios, our incorporation of multiple, driver-selected tasks offers new insights. These metrics underscore that it is not just the nature of the tasks themselves that matters but also how drivers transition between them.

This added complexity reflects real-world conditions, where drivers frequently switch their attention among various activities. Such transitions impose cognitive demands that can interfere with monitoring the driving environment and responding swiftly when takeover is required.^33^ By identifying these predictive factors, we can improve both experimental designs and the development of automated driving systems. For example, systems might adapt takeover warnings based on a driver’s current task-switching patterns, leading to safer and more responsive interactions.

In addition to examining our primary independent variable (i.e., the trial setting), we also investigated the role of time budget in shaping takeover responses. Prior research has demonstrated that drivers exhibit notably different behaviors under urgent (shorter time budget) versus non-urgent (longer time budget) conditions,^34^^,^^35^ and our findings align with these observations. In particular, some takeover performance metrics (e.g., lane change time and minimum time-to-collision) identify time budget as one of the most significant variables in our regression models. This underscores that time-related aspects of the takeover process are especially sensitive to the amount of time available, further highlighting the critical role of temporal constraints in human-machine interaction.

This study highlights the significant impact of trial settings—free-choice versus task-specific NDRTs—on driver behavior, subjective perceptions, and takeover performance in automated driving. Free-choice trials, allowing multiple task engagements and switches, led to more diverse behaviors, higher trust in automation, greater task engagement, and lower readiness to take over control. These findings reflect real-world driving complexities, underscoring the limitations of traditional single-task experimental designs. Additionally, task-switching metrics such as the number of NDRT types, switches between tasks, and timing of the last switch emerged as key predictors of takeover performance. Integrating these dynamic behaviors into research and automated system design can enhance safety and user experience. Overall, this study advances our understanding of driver interactions with automated systems and emphasizes the need for more naturalistic and multifaceted experimental approaches in future research.

### Limitations of the study

First, the use of a driving simulator, though offering a controlled and safe environment, cannot fully capture the complexity and unpredictability of on-road driving. Future research could complement simulator experiments with field tests or naturalistic driving studies to examine whether the observed patterns persist under more complex conditions.

Second, this study focused on immediate effects of trial setting and task switching on takeover performance, without exploring long-term implications. Further investigations could consider extended exposure to mixed-task scenarios, varying road or weather conditions, and individual differences in drivers’ cognitive abilities or preferences.

Third, the participant sample was predominantly male. Including a more balanced demographic profile in future studies could improve the generalizability of the findings.

Lastly, our study exclusively used the decision tree regression modeling. Although decision trees provided intuitive interpretations and were appropriate given the data characteristics, employing alternative modeling approaches, such as support vector machines or XGBoost, would further enhance the robustness and comprehensiveness of the insights regarding behavioral predictors influencing takeover performance.

In summary, future work should incorporate broader participant pools, more diverse driving contexts, and more modeling approaches and potentially integrate physiological measures or qualitative interviews to deepen our understanding of how drivers navigate multiple tasks and maintain readiness to resume control in automated driving scenarios.

## Resource availability

### Lead contact

Further information and requests for resources and reagents should be directed to and will be fulfilled by the lead contact, Qingkun Li (qingkun.li.thu@gmail.com).

### Materials availability

This study did not generate new unique reagents.

### Data and code availability

• All data reported in this paper will be shared by the lead contact upon request.
• This paper does not report original code.
• Any additional information required to reanalyze the data reported in this paper is available from the lead contact upon request.

## Acknowledgments

We sincerely thank all the participants for their efforts and feedback.

This work was supported in part by 10.13039/501100004826Beijing Natural Science Foundation (grant number 4254109), 10.13039/501100002367CAS Major Project (grant number RCJJ-145-24-14), the Tsinghua University-10.13039/501100004405Toyota Joint Research Center for AI Technology of Automated Vehicle (grant number TTAD2024-05), and the 10.13039/100009110Natural Science Foundation of Xinjiang Uygur Autonomous Region (grant number 2023D01A53).

## Author contributions

Conceptualization, W.W., Q.L., and Z.W.; methodology, W.W., Q.L., and Z.W.; investigation, Q.L., Z.W., and Y.S.; writing—original draft, Z.W.; writing—review & editing, W.W., Q.L., and Y.S.; funding acquisition, W.W., Q.L., and C.Z.; resources, W.W., C.Z., and B.C.; supervision, B.C.

## Declaration of interests

The authors declare no competing interests.

## Declaration of generative AI and AI-assisted technologies

The authors declare that no generative AI or AI-assisted technologies were used in the preparation of this work.

## STAR★Methods

### Key resources table

**Table:** 

REAGENT or RESOURCE	SOURCE	IDENTIFIER
**Software and algorithms**		

Python version 3.9.13	Python Software Foundation	https://www.python.org/downloads/release/python-3913/
Pupil Invisible Companion Software (Version 1.10)	Pupil Labs	https://pupil-labs.com/products/invisible/

**Other**		

High-fidelity driving simulator with BMW sedan on 6-DOF motion platform	Tsinghua University, State Key Laboratory of Automotive Safety and Energy	N/A
Pupil Invisible Eye Tracker (Binocular, 60 Hz, 850 nm IR)	Pupil Labs	https://pupil-labs.com/products/invisible/

### Experimental model and study participant details

#### Human participants

Thirty-two licensed drivers (27 males, 5 females) aged between 19 and 45 years (M = 28.28, SD = 8.32) participated in this study. All participants possessed normal or corrected-to-normal visual acuity. They had an average of 5.97 years of driving experience (SD = 5.25) and reported an average yearly mileage of 3515.88 km (SD = 6499.90). Participants were recruited from diverse professional backgrounds, including students, officers, and workers, to ensure a heterogeneous sample.

Prior to participation, all individuals were provided with an information sheet detailing the experimental procedures, including their right to withdraw at any time. Informed consent was obtained from all participants in accordance with the American Psychological Association Code of Ethics. The study protocol was approved by the Ethics Committee of the Department of Psychology at Tsinghua University.

### Method details

#### Apparatus

This study employed a high-fidelity driving simulator mounted on a BMW sedan on a six-degree-of-freedom motion base, as shown in Figure 1, to administer a dynamic and immersive driving experience to the participants. The apparatus was equipped with five projection screens displaying the driving environment, three of which were dedicated to the frontal view to provide a 200° field of vision. The remaining two screens were reserved for the rear view, offering a total viewing angle of 55°. This experimental configuration enabled a more authentic driving experience for the participants, as the vehicle was able to move within an angular range of ± 15° and a longitudinal range of ± 0.4 m. Throughout the simulation, various driving performance data, including speed, acceleration/deceleration, steering wheel angle, and brake pressure, were captured at a sampling rate of 60 Hz for further analysis.

To record and analyse the drivers' eye movements, a pupil invisible eye-tracker incorporating a binocular pair of infrared cameras featuring matching 850 nm infrared illuminator light-emitting diodes was employed, as depicted in Figure 2. This eye-tracking apparatus was able to precisely capture the participants' eye movements in real time, while a detachable scene camera located on the left arm of the glasses captured 1080p high-resolution scene video at 60 Hz. The gaze accuracy of the system was uncalibrated by 4.6 degrees. To facilitate a comprehensive understanding of the participants' visual engagement, Figure 3 represents the key areas of interest identified in the driving simulator environment. In this study, participants' eye movements were categorized into two primary behaviours: monitoring of driving-related areas, referred to as 'traffic', which encompasses attention to the traffic environment and dashboard, and engagement in NDRTs, specifically the use of smartphones.

#### Scenarios

The takeover scenarios involved a construction area of a long and straight three-lane highway, as illustrated in Figure 4. The ego vehicle was in autopilot mode and travelled at a speed of 90 km/h in the middle lane before the takeover request (TOR). Traffic streams were observed in the right lane, consisting of seven vehicles cruising at a constant velocity. In this situation, drivers were required to take over and merge into the right lane when the TOR was issued by the system based on the driving simulator, as depicted in Figure 5. The ego vehicle overtook the cars in the right lane, as depicted in Figure 4. The vehicles in the right lane travelled at 80 km/h with a time headway of 3.6 seconds. This headway corresponds to a distance of approximately 80 meters at 80 km/h, which is considered a realistic car-following scenario on highways while still providing sufficient space for a safe merging manoeuvre.^36^ Two types of time budgets (TBs) were implemented, i.e., 4 seconds and 8 seconds. The TBs were designed to vary the urgency of the takeover request, thereby altering the difficulty level of the takeover task. The length of each trial varied from 3 to 5 minutes to ensure that participants could not anticipate the timing of the TOR. Additionally, to better simulate actual driving conditions, the rear vehicle decelerated to prevent a rear-end collision under triggering conditions.

#### Experimental design

This study employed a within-subjects 2×2 experimental design, wherein the type of NDRT constituted the primary independent variable, while the TB was integrated to vary the difficulty level. The experimental procedure consisted of task-specific and free-choice trials conducted via smartphone applications.

Task-Specific Trials: In task-specific trials, participants were assigned to use a predesignated mobile application, such as a video, audio, gaming, or social media app, in each trial. These tasks were selected based on prior surveys indicating their prevalence in real-world automated driving scenarios.

Free-Choice Trials: In contrast, free-choice trials provided participants with the freedom to choose and switch between any of the available smartphone applications, mimicking a more naturalistic multitasking environment. Participants could engage in multiple tasks simultaneously or switch between them as they preferred.

The inclusion of varying TBs (4 s and 8 s) was strategic, aimed at preventing participants from predicting and adapting to the urgency demanded by the takeover requests.

Previous studies have explored various NDRTs, such as watching videos, playing games, and texting,^16^ identifying their extensive consumption of drivers' visual, auditory, cognitive, and motor resources.^37^^,^^38^ The literature generally supports the detrimental impact of handheld devices on takeover performance,^8^^,^^39^ as drivers exhibit a pronounced willingness to use smartphones for NDRTs.^40^ Given these insights, the inclusion of NDRT engagement, specifically via handheld devices, is crucial for evaluating its effects on takeover performance.

Our study involved an electronic survey on drivers’ willingness to engage in NDRTs on their smartphones during automated driving. The results revealed a preference for four types of apps: video, audio, gaming, and social media, aligning with previous research findings on naturalistic NDRTs. These focal app types represent a comprehensive spectrum of driver resources, including physical (e.g., use of smartphones as handheld devices), cognitive, visual, and auditory resources.

During task-specific trials, participants were limited to using one of the focal apps, reflecting the application of the designated app type as the NDRT. Contrasting these specific trials with unrestricted free-choice trials offered insights into authentic scenarios where participants engaged in NDRTs without impositions, emphasizing the study’s focus on realistic task engagement.

#### Procedures

The procedure of this study was also systematically organized to maintain coherence and reliability, as illustrated in Figure 6. Initially, a survey was administered electronically to identify the preferred NDRTs during automated driving. Following the survey, more than 50 candidates were recruited, and their demographic information was meticulously collected during their recruitment. After considering the need for age and driving experience balance, a total of 32 participants were selected for inclusion in the experiments.

The pre-experiment phase consisted of providing instructions, obtaining informed consent, calibrating the system, and then familiarizing participants with the experimental setup to ensure that they were adequately informed of and comfortable with the study scenario. During this phase, participants were informed that they would be responsible for maintaining the safety of the vehicle at all times. They were explicitly told that they were expected to take over control of the vehicle if the automated driving system issued a takeover request, and they should be prepared to do so at any moment during the trial. In particular, we showed participants the visual representation of the takeover request depicted in Figure 5 and explained that they should take over control as soon as they saw this display. Participants were also informed that NDRTs were neither prohibited nor encouraged; they had the freedom to engage in these tasks as they saw fit, while still being responsible for the safety of the vehicle.

The core of the study, the experimental phase, included 16 trials consisting of eight task-specific and eight free-choice trials. Each trial was stratified into four crucial steps: pretrial, where participants were given instructions on NDRT engagement; during-trial, where participants either engaged in NDRTs or observed the traffic; end of the trial, where participants assumed control of the vehicle; and posttrial, where participants rated their experience in terms of trust, engagement, and safety. This structured approach allowed the in-depth examination of the interactions between NDRT engagement and takeover performance in automated driving.

Randomization procedures were employed to minimize potential confounding variables that could influence the study outcomes. Specifically, the length of each trial, which varied from 3 to 5 minutes, was randomized using a diagram that was unknown to the participants and counterbalanced to ensure that participants could not anticipate the takeover request time. In addition, the time budget was distributed across the 16 trials (8 for app-type NDRT and 8 for the free-choice condition). The TB was randomized using a similar approach to the trial length, with a diagram that included 8 short (i.e., 4 s) and 8 long (i.e., 8 s) time budgets in a balanced order. This diagram was also unknown to the participants and counterbalanced across participants to control for potential order effects. To control for individual differences and mitigate potential order effects, participants were grouped based on age and driving experience. Half of the participants adopted the designated app-type NDRT first and then participated in the free-choice condition; the other half experienced these experimental conditions in the reverse order.

#### Behavioral and performance metrics

Several key indicators were derived from eye-tracking data to evaluate participants' engagement in NDRTs and attention to traffic conditions within 20 seconds before the takeover request. This time frame was selected based on typical reaction and decision-making times observed in real-world driving scenarios.^41^ The 20-second window was selected because it represents the critical period leading up to TOR, during which drivers may still be engaged in NDRTs and unaware of the impending request. This window provides an opportunity to examine how their attention and engagement with the driving task evolve as they approach the TOR.^42^^,^^43^ It allows for an assessment of whether and how NDRT engagement influences their ability to react to the TOR. Furthermore, the 20-second timeframe is sufficiently brief to ensure that the prior state of engagement does not become irrelevant to the driver’s reaction to the TOR.

Subjective self-evaluations were included to capture participants’ trust in the automated system, their perceived engagement with NDRTs, and their subjective readiness for takeover. A detailed description of these key indicators is shown in Table 1.

In addition to these eye-tracking metrics, takeover performance was assessed using simulator data, which provided more direct measures of driving performance and response to the TOR.

### Quantification and statistical analysis

All statistical analyses were conducted using Python 3.9.13. A total of 506 valid trials were included in the final analysis after excluding 6 trials due to poor eye-tracking data quality.

Descriptive statistics (medians and standard deviations) were calculated to summarize driver behaviour measures and subjective ratings. Prior to inferential testing, data distributions were examined for normality. Since the data violated the assumptions of normality required for parametric tests, the nonparametric Mann–Whitney U test was applied to compare behavioural and subjective rating differences between free-choice and task-specific trial types. Statistical significance was defined as *p* < 0.05, and all p-values reported were two-tailed. The exact n for all comparisons was 506 trials, representing individual takeover events performed by 32 participants across 16 experimental trials each.

Bootstrapped regression-based moderation analyses with 5000 bootstrap samples were conducted to explore the moderating role of trial type on the relationship between task engagement and takeover performance. This nonparametric bootstrapping approach was chosen for its robustness to violations of normality. Confidence intervals (95%) were derived using the percentile method. Moderation analyses were reported with lower and upper bounds of the confidence intervals, significance values, and effect interpretations.

Decision tree regression models were employed to further investigate the influence of behavioural variables, particularly task-switching dynamics, on takeover performance. Model performance and feature importance were visualized and reported to highlight the predictive power of these variables.

The statistical tests used, the definitions of all variables, the exact value of n, and the outcome measures (medians, U-values, p-values, and confidence intervals) are detailed in the results section, Tables 1, 2, 3, and 4, and Figures 7, 8, 9, 10, and 11.
